# RobotSDF: Implicit Morphology Modeling for the Robotic Arm

**DOI:** 10.3390/s24165248

**Published:** 2024-08-14

**Authors:** Yusheng Yang, Jiajia Liu, Hongpeng Zhou, Afimbo Reuben Kwabena, Yuqiao Zhong, Yangmin Xie

**Affiliations:** 1 School of Mechatronic Engineering and Automation, Shanghai University, Shanghai 200444, China; yysshu@shu.edu.cn (Y.Y.); jiajia@shu.edu.cn (J.L.); reubenkwabena2@shu.edu.cn (A.R.K.); ylzyq@shu.edu.cn (Y.Z.); 2 Shanghai Key Laboratory of Intelligent Manufacturing and Robotics, Shanghai University, Shanghai 200444, China; 3 Department of Computer Science, Faculty of Science and Engineering, The University of Manchester, Manchester M13 9PL, UK; hongpeng.zhou@manchester.ac.uk

**Keywords:** signed distance function, robot morphology representation, collision detection

## Abstract

The expression of robot arm morphology is a critical foundation for achieving effective motion planning and collision avoidance in robotic systems. Traditional geometry-based approaches usually suffer from the contradiction between the high demand for computing resources for fine expression and the insufficient detail expression caused by the pursuit of efficiency. The signed distance function addresses these drawbacks due to its ability to handle complex and arbitrary shapes and lower computational requirements. However, conventional robotic morphology methods based on the signed distance function often face challenges when the robot moves dynamically, since robots with different postures are modeled as independent individuals but the postures of robots are infinite. In this paper, we introduce RobotSDF, an implicit morphology modeling approach that can express the robot shape of arbitrary posture precisely. Instead of depicting a whole model of the robot arm, RobotSDF models the robot morphology as integrated implicit joint models driven by joint configurations. In this approach, the dynamic shape change process of the robot is converted into the coordinate transformations of query points within each joint’s coordinate system. Experimental results with the Elfin robot demonstrate that RobotSDF can accurately depict robot shapes across different postures up to the millimeter level, which exhibits 38.65% and 66.24% improvement over the Neural-JSDF and configuration space distance field algorithms, respectively, in representing robot morphology. We further verified the efficiency of RobotSDF through collision avoidance in both simulation and actual human–robot collaboration experiments.

## 1. Introduction

The morphology of a robotic arm refers to the way in which the shape, structure, and configuration of the robot arm are represented and described [1]. This morphology is crucial for the robot to execute various functions such as obstacle avoidance trajectory planning in human–robot interaction [2], human–robot collaboration [3], and digital twin applications [4,5]. Generally, the robotic arm is represented as unions of simple geometric shapes, like cylinders [6,7], spheres [8,9], capsules [10,11], voxels [12,13], etc. However, geometry-based description exhibits certain limitations that stem from their reliance on explicit geometric models. For example, geometry-based robotic morphology loses the detail of the robotic shape and can increase the likelihood of false positives in collision detection scenarios [14].

Instead of explicitly expressing the robot’s body shape based on geometric primitives, some researchers tend to express the morphology of the robotic arm through implicit functions. The implicit expression of the robot indirectly outlines the robot’s contour in space by utilizing the distance from other points in space to the robot’s surface, with the signed distance function (SDF) being the most popular method [15,16,17]. The SDF of a robotic arm can be regarded as a shape-based classifier, where the classification boundary corresponds to the robot’s surface [18,19]. For any query point, the SDF value represents the shortest distance from the query point in space to the robot surface, in which a positive value indicates that the point is located outside the robotic arm, and a negative value indicates that it is inside the robotic arm. SDF values for the points that lie on the surface of the robotic arm are zeros. One classic morphology approach based on SDF is called DeepSDF [20], which successfully applied this technique to represent a wide range of objects. However, DeepSDF can only represent static objects with fixed posture. Therefore, when the robotic arm changes its configuration due to joint motions, the surface geometry, if using DeepSDF, needs to be considered as a new object and retrained. The combination of the robot joint angles is infinite; therefore, it is not feasible to train a DeepSDF model for each robot status. Some researchers have attempted to solve this problem. The related works involve the visual self-modeling procedure [21] and the neural joint space implicit SDF [22]. However, these methods usually model the robotic arm as a complete object, which makes the overall model highly sensitive to the robot’s posture parameters. Additionally, their reconstruction accuracies based on SDF values are limited, particularly at the joint connection areas, where noise and errors are prone to occur.

In this paper, we propose a network-based implicit morphology method for robotic arm morphology called “RobotSDF”, which is capable of representing the continuous dynamic postures of the robot, offering a framework for accurately modeling robot morphology in different configurations. The inputs for RobotSDF include the joint configuration of the robot and the coordinates of query points, and the output data are the corresponding SDF values. In this approach, RobotSDF encodes the shape of the robot as a continuous function that can answer space occupancy queries given the current robot configuration. Specifically, rather than depicting a whole model of the robotic arm, RobotSDF models the robot morphology as integrated implicit joint models driven by joint angles. The foundational concept of RobotSDF is that the shape of each joint remains constant in the process of changing the robot’s posture, while the relative spatial relationship of the joints changes. RobotSDF converts the process of the robot morphology change into the coordinate transformation of query points within each joint’s coordinate system. This transformation simplifies the difficulty of modeling the entire robot shape and improves the calculation efficiency.

**Figure 1:**
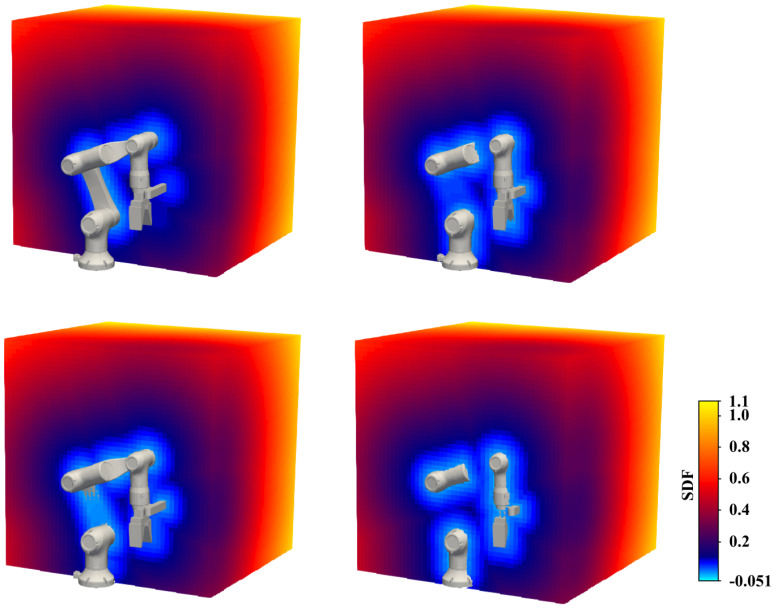
The example of the proposed SDF in one posture. The SDF values in different slices are given, and the colors from blue to yellow represent the robot’s SDF distance from near to far.

As shown in Figure 1, given a spatial point coordinate, the SDF value of a posture-known robot can be produced by RobotSDF directly, and the full shape of the robot can be discretized for visualization by methods like Marching Cubes [23]. This capability allows RobotSDF to be applied in various domains, such as collision detection. A positive signed distance means no collision, and a negative or zero signed distance expresses that collision happens. RobotSDF not only facilitates collision detection but also enables a nuanced evaluation of the proximity to potential collisions, thereby mitigating the risk of collision before it happens. Since RobotSDF can sense the shortest distance from any position in the field to the robot, it can respond quickly to irregularly shaped objects or deformable obstacles. The main contributions of this paper include:

- We propose an implicit modeling method for continuous robotic morphology, called RobotSDF, by converting the robot’s overall morphology changing process into the transformation process of query points within the local coordinate of each joint.
- We verify the morphological expression accuracy of the proposed RobotSDF on a six-DOF robotic arm, which can achieve high reconstruction accuracy up to the millimeter level.
- We propose a collision detection strategy based on the proposed RobotSDF and verify the effectiveness and efficiency of the algorithm in the simulation and real manipulation experiments.

The organization of the paper is as follows. In Section 2, a brief review of related work about morphology modeling of robots is introduced. The Section 3 outlines the methodology of RobotSDF construction. The implementation details about RobotSDF are given in Section 4. Then, the experiments with RobotSDF regarding morphology accuracy are evaluated in Section 5, as well as the collision detection experiments based on RobotSDF in simulation and real manipulation tasks. Section 6 concludes the paper and produces the direction of future work.

## 2. Literature Review

Morphology modeling methods for robots can be roughly divided into two categories: geometry-based representation and SDF-based representation. The former approach involves combining simple geometric shapes to approximate the shape of the robot. The latter implicitly represents the robot’s shape by utilizing the distance from points in space to the surface of the robot.

### 2.1. Geometry-Based Morphology Modeling

Geometry-based morphology modeling is a widely adopted approach in the field of robotics that is characterized by the use of explicit geometric shapes to represent the physical structure of a robot [24]. This method relies on the assembly of simple geometric primitives, such as cubes, spheres, cylinders, and other basic shapes, in order to construct a composite form that approximates the desired morphology of the robot.

The sphere is one of the geometric shapes most widely used to represent the robot’s form due to its computational efficiency [25,26]. By adjusting the radius of the sphere, varying degrees of precision in shape approximation can be achieved, allowing for flexible and scalable simulations. For example, Tomáš Kot et al. [27] employed a multi-sphere representation to model the irregularly shaped body of a robotic arm and performed obstacle avoidance within a static environment. To prevent the self-collision of a dual-arm robot, Maolin Lei et al. [28] built a collision model using discretized finite spherical volumes with the same radii at specific positions. Similar approaches were used by Roberto Simoni et al. [29], who adopted three safety spheres to fully wrap the I-AUV equipment.

The cylinder is another geometric shape frequently employed to represent the shape of a robotic arm in a simple manner, particularly due to its resemblance to robot links [30]. As presented in [6], Guanglong Du et al. used cylinders to establish the bounding box model for human bones and robots and conducted the collision detection between cylinders during human–robot collaboration. Shuangning Lu et al. [31] took the cylinder bounding box as the bounding box of the robotic link and the sphere bounding box as the bounding box of the robotic joint, and they solved the problem of collision detection in the process of human–robot collaboration as well.

The capsule shape, a variation of the cylinder, combines a cylindrical body with hemispherical ends. This hybrid geometry allows it to better accommodate situations where the end faces of robot joints are non-planar, offering a more accurate and adaptable representation. For example, in the human–robot collision avoidance task, Mohammad Safeea et al. [32] represented the human coworker and the robot by the capsules at the same time and calculated the minimum distance and relative velocity among them during the collaboration. A similar strategy of collision avoidance for human–robot interaction was proposed by Mohammad Safeea and Pedro Neto [33], who represented the human with five capsules and represented the robot with three capsules.

In addition to the sphere, cylinder, and capsule shapes, polygons can also be employed to simplify the representation of a robotic arm [34,35]. However, morphology modeling of the robotic arm based on simple geometric shapes often encounters issues with the omission of details. In scenarios requiring precise operations, this lack of detail could lead to incorrect collision detection judgments, potentially causing tasks that can be achievable to be mistakenly deemed as impossible.

### 2.2. SDF-Based Morphology Modeling

The utilization of SDF for shape representation is an active research direction in robotics due to its ability to handle complex environmental shapes, including the obstacles and the robot [15,20,36,37,38]. For example, a continuous implicit SDF model was presented by Tingrui Zhang et al. [39] for trajectory optimization, and the corresponding SDF model is suitable for a robot of any shape.

In terms of an implicit SDF model for representing the robotic arm, Boyuan Chen et al. [21] proposed a body self-modeling approach based on SDF for robot morphology representation. Specifically, they used a single implicit neural network to answer space occupancy queries given the current posture or the possible further states of the robot. Mikhail Koptev et al. [22] proposed the Neural-JSDF, which is a neural joint space implicit SDF for a given robot, and extended its application to a quadratic program inverse kinematics solver and sampling-based model predictive controller. Yiming Li et al. [40] employed a combination of Bernstein polynomials to encode the signed distance for each robot link and leverage the kinematic chain to produce the SDF representation in joint space. Mark Nicholas Finean et al. [41] explored the use of composite SDF in motion planning and how they can be used in predicting obstacle motions. Puze Liu et al. [42] proposed the regularized deep SDF that can compute a smooth distance field at any scale and verified its effectiveness in human–robot interaction experiments. Ming-Hsiu Lee and Jing-Sin Liu [43] constructed an SDF to describe the swept volume of obstacles to identify the safe protective space for operation.

The above research shows the feasibility of using SDF to represent the robot’s morphology. However, current SDF-based methodologies usually encounter the challenge of limited accuracy of morphological representation, which is particularly pronounced when the robot moves dynamically.

## 3. Methodology

In this section, we propose RobotSDF, an implicit continuous robot morphology modeling approach for collision detection. An SDF is a continuous field in which every point in space is associated with its minimum distance to an object’s surface, and the corresponding zero isosurface refers to the object surface boundary [20]:

(1) $$SDF\left(P_{w}\right)=\left\{d_{w}|P_{w}\in R^{3},d_{w}\in R\right\},$$

where $P_{w}$ refers to the point position in the world coordinate, and $d_{w}$ means the minimum distance from $P_{w}$ to the object’s zero surface. Let $Sign(d_{w})$ denote the sign of $d_{w}$, in which a negative sign(−) indicates that $P_{w}$ is located inside the object, and a positive sign(+) implies that $P_{w}$ is located outside the object’s surface. When $d_{w}$ equals zero, $P_{w}$ is placed on the object’s surface.

(2) $$\left\{\begin{array}{cc} P_{w}\;\mathrm{is}\;\mathrm{inside} & \;\mathrm{if}\;Sign(d_{w})<0\\ P_{w}\;\mathrm{is}\;\mathrm{on}\;\mathrm{the}\;\mathrm{surface} & \;\mathrm{if}\;d_{w}=0\\ P_{w}\;\mathrm{is}\;\mathrm{outside} & \;\mathrm{if}\;Sign(d_{w})>0 \end{array}\right.$$

Conventional SDF is typically applicable solely to static objects without consideration for dynamic variations. However, the distance from a specific point in the world coordinate system to the robot’s surface would change as the robot’s configuration changes. Therefore, we introduce a novel concept termed RobotSDF, specifically devised to articulate the SDF value relative to the dynamic robot surface. Specifically, RobotSDF extends the conventional SDF by incorporating the robotic configuration parameters, thereby accounting for the dynamic properties of the robot’s morphology:

(3) $$RobotSDF\left(P_{w},Q\right)=\left\{d_{w}^{Q}|P_{w}\in R^{3},d_{w}\in R,Q\in R^{N}\right\},$$

where $Q=\{q_{1},\cdots ,q_{N}\}$ represents robotic joint angles, *N* denotes the joint degrees of freedom, and $d_{w}^{Q}$ is the minimum distance from point $P_{w}$ to the robot in configuration *Q*.

**Figure 2:**
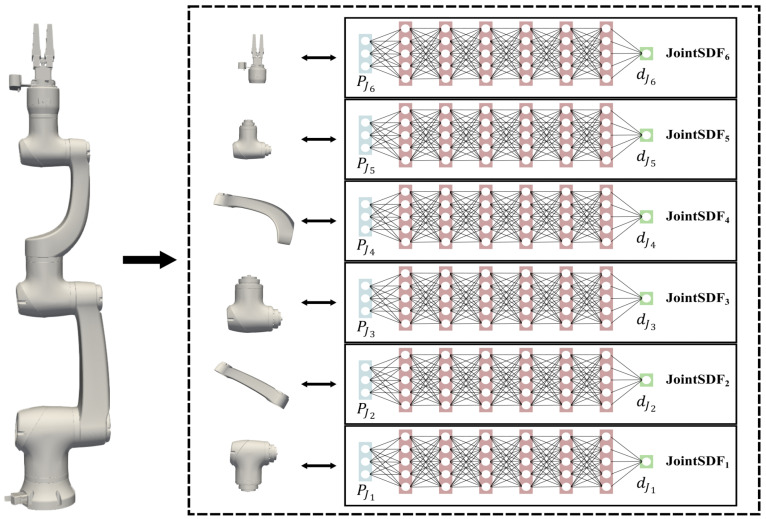
The robot model is separated into several independent joint links, and each link is implicitly represented as a neural network, which is regarded as the JointSDF. The input of RobotSDF is the position of the spatial point in the joint local coordinate, and the output is the corresponding SDF value to the joint.

Although it is feasible to represent robotic morphology implicitly using an SDF neural network, with inputs comprising the point coordinates and joint angles and output yielding the corresponding SDF value, it is notable that the accuracy of the robotic model, which is reconstructed by the network, tends to be compromised, particularly in regions near joint links [21]. In this paper, we segment the robot model into several independent joint links and leverage implicit neural representation, which is regarded as the JointSDF, to model each joint link, as shown in Figure 2. Specially, to facilitate practical application, the gripper and the end joint are treated as one in this paper, since they are usually fixed together.

For a given spatial point, its position in the world coordinate system is transformed into the local coordinate system of each joint, and corresponding SDF values regarding each joint can be obtained based on JointSDFs accordingly:

(4) $$JointSDF\left(P_{J_{n}}\right)=\left\{d_{J_{n}}|P_{J_{n}}\in R^{3},d_{J_{n}}\in R\right\},$$

where $P_{J_{n}}$ is the point position in the $n_{th}$ local joint coordinate, and $d_{J_{n}}$ is the minimum distance to the corresponding joint.

In this approach, the morphology deformation process is converted into the position transformation process of the query point within the local coordinate system of each robot joint. It is important to note that the joint itself can be considered a static object during the transformation.

In the following, we explain the methodology for constructing the JointSDF and subsequently combining JointSDFs to form RobotSDF. Furthermore, we introduce the collision detection approach by leveraging the RobotSDF representation.

### 3.1. JointSDF

**Figure 3:**
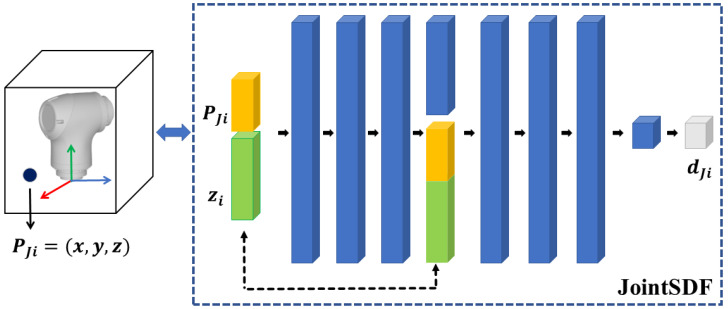
The structure of JointSDF. The inputs are the position $P_{J_{i}}$ in the local coordinate system and the latent vector $z_{i}$ of the joint. The output is the corresponding SDF value $d_{J_{i}}$.

As mentioned above, the JointSDF only describes the static shape of a single joint, and each joint needs to be described separately. Many implicit representation methods for static 3D shapes have been proposed in recent years, and DeepSDF is a popular one owing to its efficiency and effectiveness [20], as it encodes the object shape as a latent vector in 256 dimensions. JointSDF adopts the same structure as DeepSDF to model the joint shape, as shown in Figure 3. Specifically, JointSDF encodes the joint shape as the joint latent vector and predicts the SDF value by combining the query point position $P_{J}$. Formally, JointSDF is a feed-forward network composed of eight fully connected layers, with the output regressed as the desired SDF value. The training process involves minimizing the sum over the disparities between predicted and actual SDF values of points within the training set. It is important to note that the ground truth of sample positions and corresponding SDF values are conducted within the local coordinate system of the respective joint. The corresponding latent vector for each joint can be estimated based on maximum a posteriori (MAP). Upon completion of training the latent vector of the joint, the latent vector remains unchanged throughout the regression process.

### 3.2. RobotSDF

**Figure 4:**
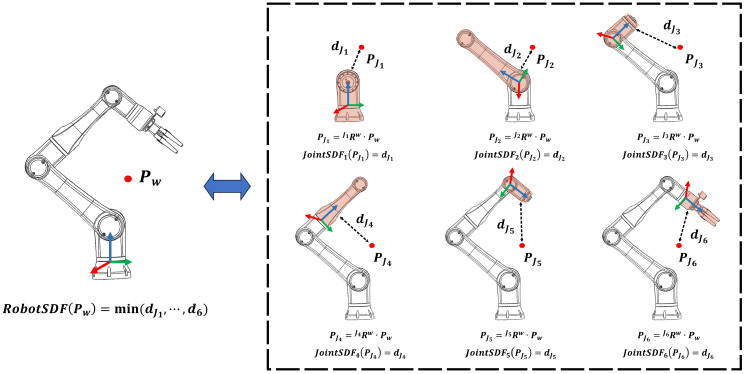
The calculation procedure of RobotSDF.

For a query point $P_{w}$ in the world coordinate system, the corresponding RobotSDF represents its minimum distance to the robot surface, i.e., the minimum SDF value among all JointSDFs. As illustrated in Figure 4, the local position $P_{J_{i}}$ for $P_{w}$ in the $i_{th}$ joint coordinate can be calculated as follows:

(5) $$P_{J_{i}}={\;}^{J_{i}}R^{w}\cdot P_{w},$$

where${\;}^{J_{i}}R^{w}$ denotes the transformation matrix from the world coordinate to the $i_{th}$ joint’s local coordinate, which can be computed according to forward kinematics. The corresponding JointSDF value is calculated as:

(6) $$JointSDF\left(P_{J_{i}}\right)=JointSDF{(}^{J_{i}}R^{w}\cdot P_{w}).$$

Eventually, the RobotSDF regarding the specific robotic posture *Q* can be calculated as:

(7) $$\begin{array}{cc} \; & RobotSDF\left(P_{w},Q\right)=d_{w}^{Q}\\ \; & =\{min\left(JointSDF{(}^{J_{i}}R{\left(q_{1,\cdots ,i}\right)}^{w}\cdot P_{w})\right)|i=1\cdots N\}. \end{array}$$

**Figure 5:**
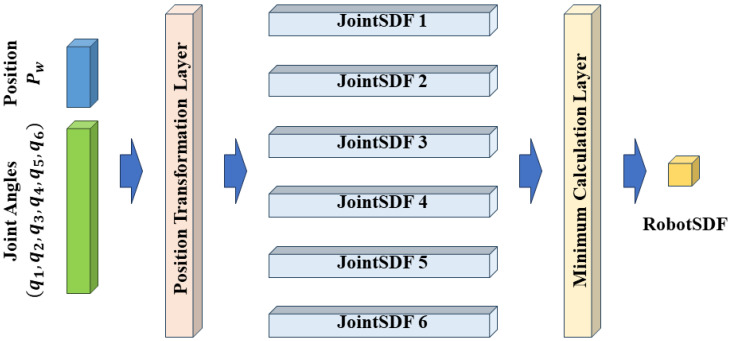
The network structure of RobotSDF.

We further format the calculation procedure of RobotSDF in the network structure, as shown in Figure 5, which consists of the input layer, position transformation layer, JointSDF layer, minimum calculation layer, and the output layer. By vectorizing the network input, the network can simultaneously calculate RobotSDF values for multiple query points, greatly improving the computing efficiency in practical applications.

### 3.3. Collision Detection Based on RobotSDF

For a single point $P_{w}$, a collision occurred if $d_{w}^{Q}\leq 0$, otherwise $P_{w}$ does not collide with the robot. In practice, for irregular shape obstacles, we uniformly sample points on the obstacle surface and check the collision state of all samples:

(8) $$\mathrm{Collision}=\left\{\begin{array}{cc} True & \;\mathrm{if}\;\left\{Any(d_{w_{k}}^{Q}<0)|k=1,\cdots ,K\right\}\\ False & \;\mathrm{Otherwise}\; \end{array}\right.,$$

where *K* represents the number of sample points for the target obstacle. If all sample points are not in the collision state, the obstacle does not conflict with the robot. Otherwise, the obstacle is regarded as colliding with the robot if any sample point is in a collision state.

## 4. Implementation Details

### 4.1. Data Preparation

In this paper, the Elfin-3 collaborative from Han’s Robot Company is adopted as the platform for building the RobotSDF network. Accurate and sufficient training data are essential to train the RobotSDF network. As the inputs for the RobotSDF include the coordinates of the query point and joint angles, and the output is the corresponding SDF value, each training sample should comprise the coordinates of the sampling point, the joint angle, and the corresponding SDF value. In this paper, robot models in various postures are generated and represented using a high-fidelity STL format. Each model contains about 492,000 vertices and 983,000 faces, with a corresponding file size of about 47 megabytes, which can describe the robot shape in detail. Then, we sample query points in 3D space and calculate the SDF values from sampling points to the robot model based on the MATLAB Robotic Toolbox. Since RobotSDF contains several independent JointSDFs, sample data should be prepared for each joint respectively.

**Figure 6:**
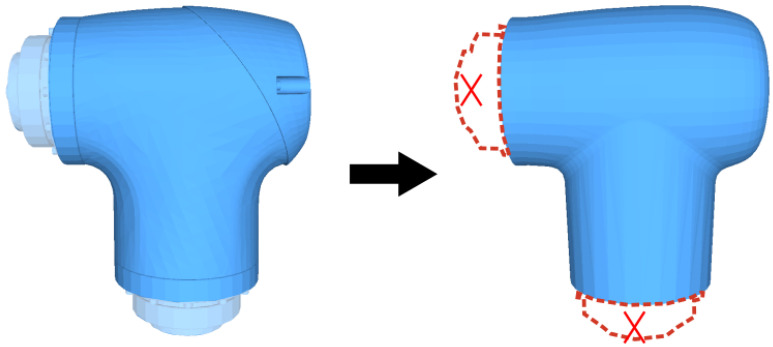
During the sampling procedure, the 3D model of the robot joint is simplified by omitting the interconnection structure and replacing it with a plane.

**Figure 7:**
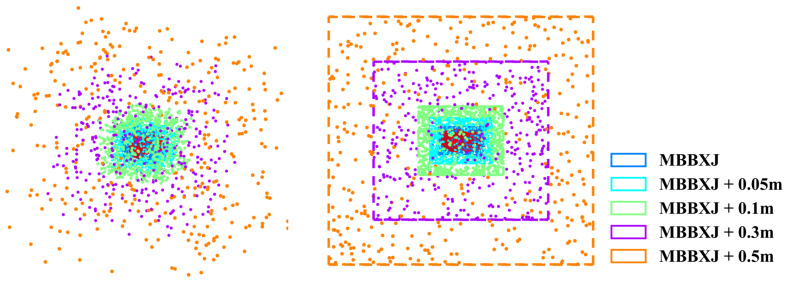
The sampling space for each joint is divided into five regions. The first region is the inside space of MBBXJ. The next four regions diffuse outward from the MBBXJ surface by distances of 0.05 m, 0.1 m, 0.3 m, and 0.5 m, separately.

As data quality is critical to network performance, several principles are followed during the sampling procedure: (1) The joint model is simplified before sampling to reduce the complexity of SDF calculation. As shown in Figure 6, the intricate interconnection structure between joint components has been substituted with a planar structure, as this segment is covered by the adjacent joint and remains unobservable during the manipulation. (2) The sampling points are positioned within a range from the center of the minimum bounding box of the joint (MBBXJ) spread to a certain threshold. Because there is no collision when the sampling point lies beyond the robot’s workspace, the necessity to access collision detection is obviated. (3) The likelihood of the collision increases significantly as the SDF distance approaches the joint surface. Therefore, more sampling points are allocated near the joint surface to improve prediction accuracy. As shown in Figure 7, the sampling space is divided into five regions, and for each region, the points are sampled uniformly. The initial region extends from the center of MBBXJ to the MBBXJ surface. The subsequent four regions diffuse outward from the MBBXJ surface by distances of 0.05 m, 0.1 m, 0.3 m, and 0.5 m separately. The distribution of sampling points in these five regions relative to the total number of sampling points are 30%, 30%, 20%, 10%, and 10%, respectively. Eventually, 2.5 million points are sampled randomly for each joint, and 80% of the sampling points are designated for training, while the remaining 20% are allocated for testing.

### 4.2. Network Training

To improve training efficiency, all JointSDFs are trained with the same strategy. For each JointSDF, the optimization targets include the network parameters and the joint’s latent shape code. For both trainings, the optimizer is set as Adam, and the dropout probability is set as 0.2. We set the batch size as 32 with 2000 epochs. For network parameter training, the initial learning rate is 0.0005, and it decays by multiplying a factor of 0.5 every 500 steps. During the training of latent shape code, an initial learning rate of 0.001 is employed, undergoing the same adjustment as network parameter training.

## 5. Experiments

In this section, we explain three experiments that were conducted to evaluate the performance of the proposed RobotSDF from different aspects. Experiment 1 verified the reconstruction accuracy of RobotSDF regarding different postures. Experiment 2 evaluated the collision detection accuracy by comparing RobotSDF with conventional approaches. The adaptability of RobotSDF was estimated in Experiment 3 by integrating it with the path planning algorithm. All experiments were conducted using a computer embedded with the Intel^®^ Xeon^®^ Platinum 8124 CPU and an NVIDIA GeForce RTX 3090 Ti GPU. The network of JointSDF is implemented based on the PyTorch framework, with Han’s Elfin-3 collaborative robot serving as the designated platform.

### 5.1. Reconstruction Accuracy of RobotSDF

#### 5.1.1. JointSDF Evaluation

In this experiment, the ability of JointSDF for each robotic joint is verified based on the root mean square error (RMSE) between the true SDF value and the predicted value based on the corresponding JointSDF. The formula for RMSE calculation for the $j_{th}$ joint is given as follows:

(9) $$RMSE_{j}=\sqrt{\frac{\sum_{i=1}^{N}{\left(d_{j}-\hat{d_{j}}\right)}^{2}}{N}},$$

where $RMSE_{j}$ means the RMSE error for the $j_{th}$ joint, and *N* is the number of sampling points during the evaluation. Table 1 presents the evaluation results of the JointSDF network predictions based on 30,000 sampling points. The minimum RMSE observed is 5.13 mm for JointSDF2 with a minimum standard deviation of 3.17 mm, while the maximum RMSE is 8.11 mm for JointSDF1, which also has a maximum standard deviation of 7.58 mm.

Table 2 presents a comparative analysis of the average RMSE between the proposed RobotSDF and Neural-JSDF proposed by Mikhail Koptev et al. [22] and the configuration space distance field (CDF) [44]. Neural-JSDF is employed to construct an SDF model of the Franka Emika Panda robot, yielding an average RMSE of 10.40 mm and a standard deviation of 7.80 mm for all joints. Compared to Neural-JSDF, the proposed RobotSDF demonstrates an improved performance, with an average RMSE of 6.38 mm and a standard deviation of 4.62 mm. This represents a 38.65% reduction in average error compared to Neural-JSDF. Additionally, even the joint with the highest RMSE in the RobotSDF exhibits a 22.02% lower error than Neural-JSDF. Similarly, when representing the morphology of the Franka robot, the RMSE achieved by CDF reaches 18.90 mm, associated with a standard deviation of 8.00 mm. Compared with CDF, the accuracy of the proposed RobotSDF is improved by 66.24%.The experimental results show that RobotSDF has higher accuracy in expressing the joint morphology of the robot.

#### 5.1.2. RobotSDF Evaluation

To assess the fidelity of RobotSDF in representing the morphological variations across various robotic configurations, we sampled 1012 sets of joint angles randomly from the robot’s state space and quantified the dissimilarity between the 3D models reconstructed by RobotSDF and the corresponding ground truth by measuring the Chamfer Distance (CD).

**Figure 8:**
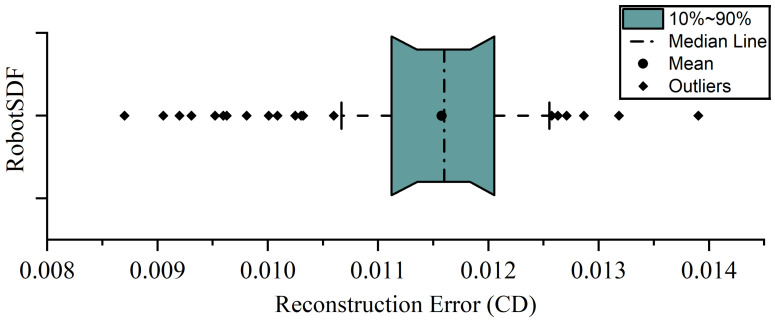
The distribution of CD error in the RobotSDF reconstruction experiment.

Figure 8 depicts the distribution of CD in a box plot, which reveals a mean CD of 0.01158 and a median CD of 0.01160. For 80% of the samples, the CD error falls within a narrow range, specifically between 0.0111 and 0.0121, indicating a consistent level of approximation across the sampled postures. Among all samples, the maximum CD error is 0.0139, and the minimum CD error is 0.0087.

**Figure 9:**
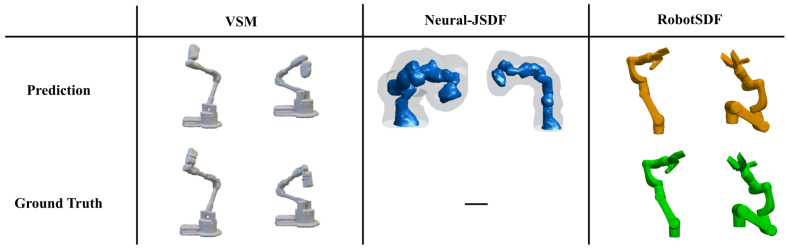
The comparison of robot morphology expression capabilities among three algorithms (VSM, Neural-JSDF, and RobotSDF). The pictures of VSM [21] and Neural-JSDF [22] are from corresponding papers. Since the ground truth picture of the Neural-JSDF is not given in their paper, only the prediction result is given here. The proposed RobotSDF shows the smoothest and most detailed robot surface.

**Figure 10:**
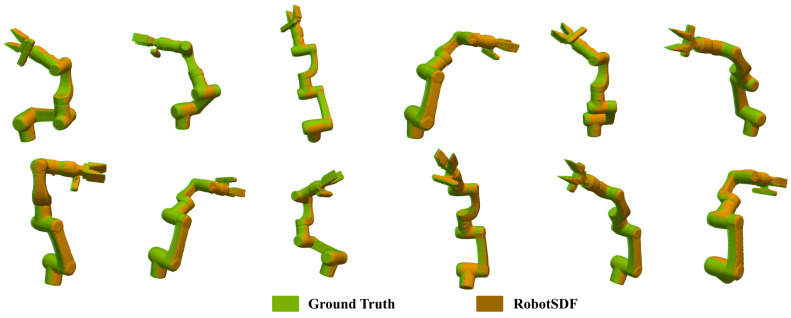
The comparison between RobotSDF and the ground truth. The visualization of the RobotSDF model is drawn in orange, and the corresponding ground truths of the robot are colored green.

The experimental results underscore the ability of RobotSDF to adeptly generate 3D robot models across a diverse range of postures with high accuracy. Figure 9 illustrates the comparison of robot morphology expression capabilities among the Visual Self-Modeling (VSM), Neural-JSDF, and RobotSDF separately. It is obvious that RobotSDF shows the smoothest and most detailed robot surface. More reconstruction results of RobotSDF are shown in Figure 10 to provide visual representations of the accuracy.

### 5.2. Collision Detection Evaluation

**Figure 11:**
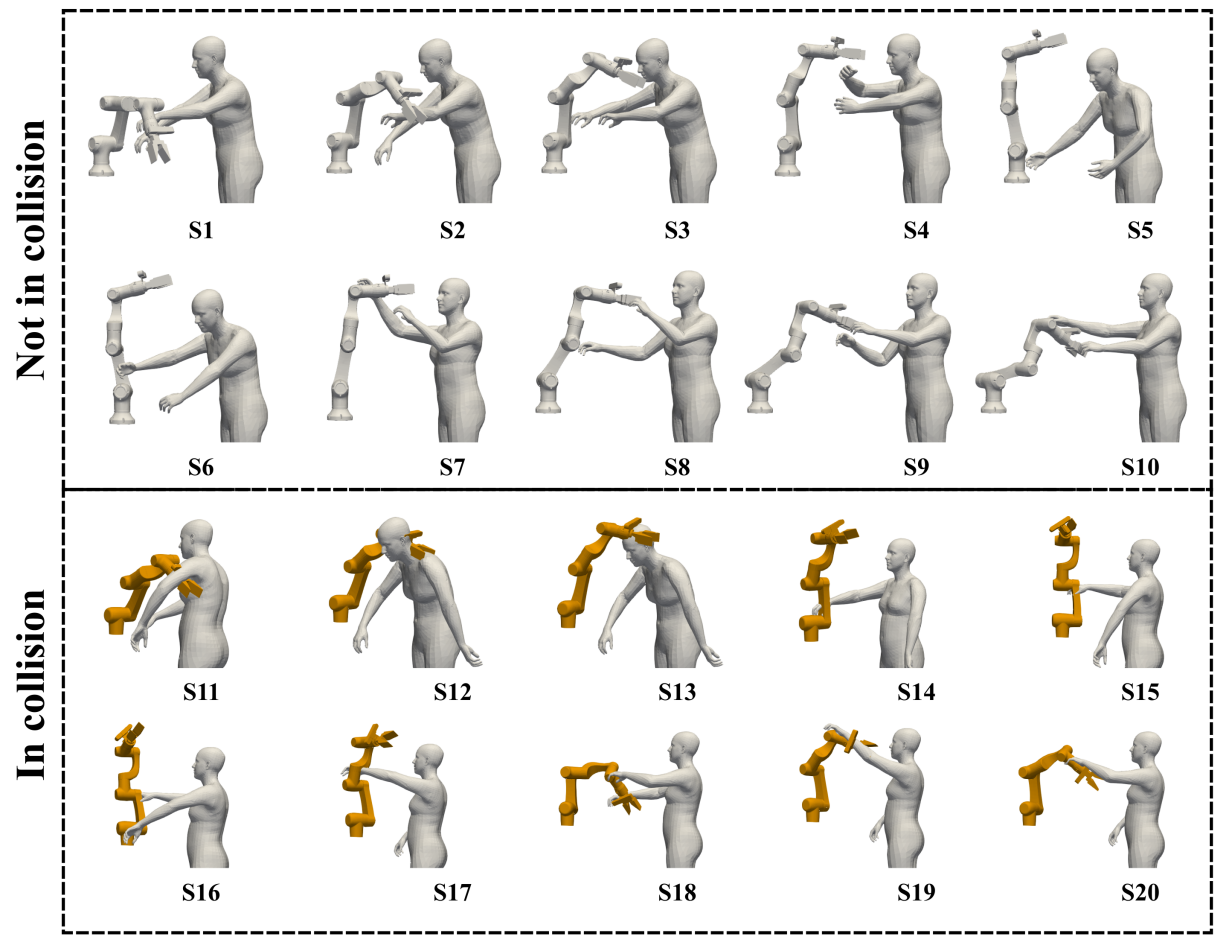
Twenty scenarios are generated to evaluate the collision detection accuracy of RobotSDF. The ground truths of S1 to S10 are not in collision, and the ground truths of S11 to S20 are in collision.

To evaluate the collision detection efficacy of RobotSDF in the task of human–robot collaboration, 20 distinct scenarios were simulated as shown in Figure 11, and the corresponding collision detection results were compared with two classic algorithms: the Separating Axis Theorem (SAT) [45] and the Gilbert–Johnson–Keerthi (GJK) algorithm [46]. The human body in the simulation is randomly generated based on the SMPL model [47]. In the first 10 scenarios (S1–S10), the human model does not physically intersect with the robot, and human models intersect with the robot in the last 10 scenarios (S11–S20).

During the process of collision detection, the 3D mesh models of the robot and the human body are utilized as inputs for three algorithms. For SAT and GJK, the robot model needs to be separated into six segments according to joints, and the human body model is divided into three segments based on the torso, left arm, and right arm. Subsequently, the polygonal convex is generated for each segment based on the FCL library, and collision detection is conducted between all robot joint convexes and all human body segments. Therefore, the collision detection time of GJK and SAT includes the model segmentation time, convex generation time, and convex collision detection time. In contrast, the RobotSDF model allows for a more streamlined approach, which can process the vertices of the human body model (about 10,000 vertices) by treating them as network inputs and which yields the collision detection result directly. This direct input approach enhances the efficiency of the detection process of the RobotSDF compared to SAT and GJK.

Table 3 summarizes the collision detection results for three algorithms, and the ground truths (GTs) are given as well. For scenarios from S11 to S20, all three algorithms can precisely identify the collision results. For scenarios S1 to S10, RobotSDF accurately discerns that there are no collisions between the robot and the human model. However, SAT and GJK misidentified some cases, such as S1, S3, S6, and S10 for SAT and S1 for GJK. In scenario S1, both SAT and GJK erroneously infer a collision when the human’s left arm is positioned within the robot’s joint angle and close to the joint. These errors arise because both SAT and GJK tend to inflate the object’s contour during collision detection. Additionally, SAT exhibits false detections in scenarios S3, S6, and S10, attributed to the human’s proximity to the robot. The experimental results underscore RobotSDF’s ability to precisely detect collision status during human–robot collaboration, mitigating the risk of abrupt shutdown of the robot.

**Figure 12:**
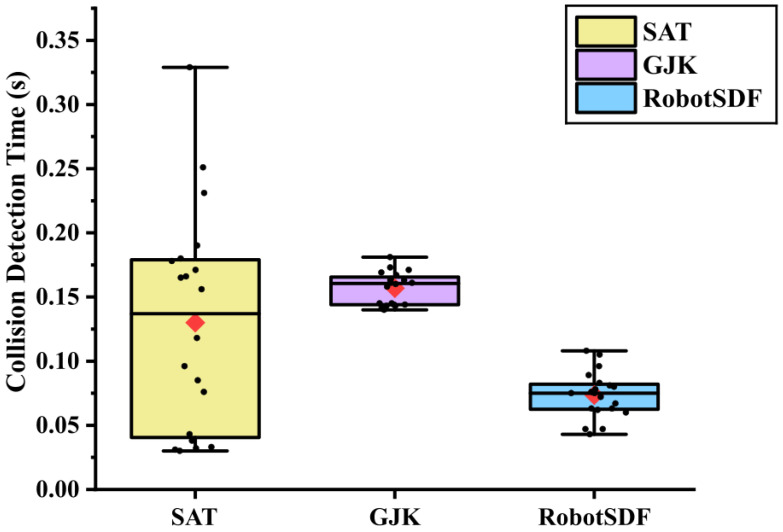
The collision detection time performance of SAT, GJK, and RobotSDF in experimental scenarios. The collision detection time for SAT and GJK includes the model segmentation time, convex generation time, and convex collision detection time. RobotSDF can process the collision detection by taking the vertices of the human body as RobotSDF network inputs, and it yields the collision detection results directly.

In addition to the collision detection accuracy, the collision detection efficiency of RobotSDF was compared with GJK and SAT as well, as shown in Figure 12. The mean collision detection times for SAT, GJK, and RobotSDF were 0.1299 s, 0.1568 s, and 0.0735 s respectively, and the associated variances were 0.0069 s, 0.0001 s, and 0.0003 s. Overall, RobotSDF exhibits remarkable detection efficiency, capable of detection at the centisecond level, surpassing the performance of SAT and GJK, which achieves detection efficiency at the decisecond level. In terms of detection stability, RobotSDF and GJK perform similarly with small variances, and a higher variance occurs for the SAT algorithm. That may be because numerous query points can be taken as inputs to the RobotSDF network at the same time, and the detection results can be identified simultaneously. Processing a substantial amount of data concurrently enables RobotSDF’s rapid collision detection ability even in environments with various obstacles. Experimental results demonstrate that RobotSDF can accurately identify collisions in confined environments, including those involving highly irregular shapes such as the human body. This precision is maintained even when the human performs complex postures at a close distance from the robot, highlighting the effectiveness of RobotSDF in collision detection and enhancing the robot’s operational capabilities in restricted spaces.

The primary drawback of RobotSDF is the necessity for extensive data sampling during the network training process to achieve a high-precision model, which could result in significant training time. For example, it takes about one month to train RobotSDF using an NVIDIA 3090 GPU card.

### 5.3. Adaptability Evaluation

**Figure 13:**
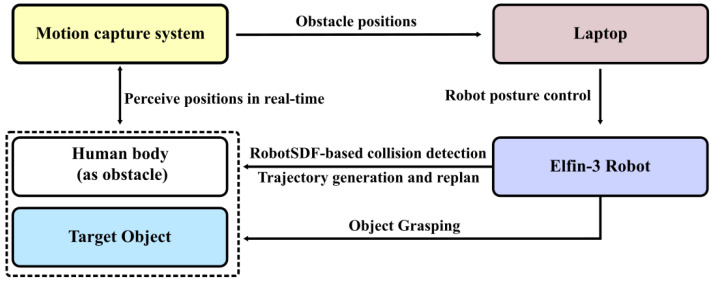
The structure of the experimental system.

Two human–robot intersection experiments were conducted within the real-world environment to evaluate the practical applicability of RobotSDF. As shown in Figure 13, the experimental system comprises the robot body, a target object placed on a table, a laptop as the control center, a person as the obstacle, and the motion capture system. Markers are affixed to both the target object and the human body, allowing the motion capture system to sense their spatial positions. The motion capture system and the robot are connected to the laptop via the network cable, enabling real-time data interaction. During the experiment, the robot perceives the spatial positions of the human body in real time and checks if they are in collision with the robot. The trajectory of the robot to the target object is first generated based on the Rapidly-exploring Random Tree (RRT) algorithm, and the robot follows accordingly. Then, the system will determine whether the subsequent trajectory will collide with the person; if so, the trajectory will be re-planned until a new collision-free trajectory is built.

**Figure 14:**
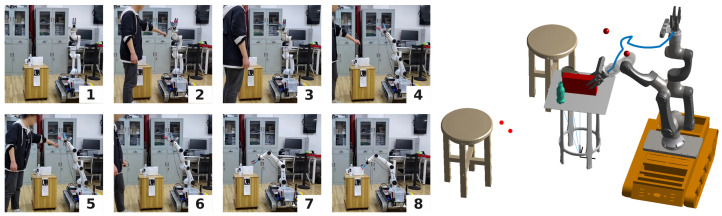
The procedure of collision detection, Experiment 1. The target object for the robot’s grasping task is a cube. During the manipulation process, the human arm acts as an obstacle. When the robot senses the obstacle based on the RobotSDF model, the robot will re-plan the trajectory.

**Figure 15:**
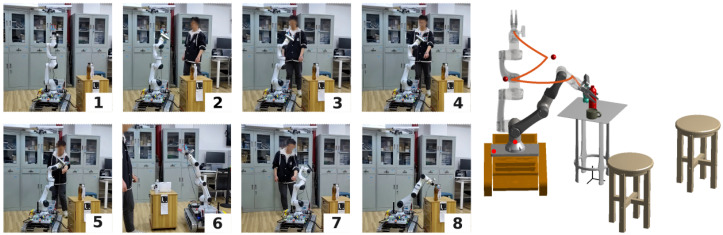
The procedure of collision detection, Experiment 2. The procedure of Experiment 2 is similar to the procedure of Experiment 1; however, the initial postures of the robot change, and the target object is replaced with a bottle.

In both experiments, the robot navigated from its initial posture to the designated grasping posture, and the operator interrupted the original trajectory. Figure 14 and Figure 15 depict the experimental procedure, and the trajectories of the robot’s end effector are highlighted to illustrate the trajectory-changing process. The red ball in the figure symbolizes the position of the detected obstacle. It is obvious that, when the obstacle remains at a distance from the robotic arm, RobotSDF detects the potential collision along the forthcoming trajectory and promptly adjusts the robot’s path accordingly. The full video can be watched at the following link: https://youtu.be/atN8O9wckTM (accessed on 10 July 2024).

## 6. Conclusions

In this paper, we presented an implicit expression of robot morphology, called RobotSDF, that can represent the shape of the robot in any posture. The proposed RobotSDF can capture the minimum SDF value of the query point within the working space and determine the collision status based on the sign of the SDF value. RobotSDF offers distinct advantages in scenarios involving intricate deformable obstacles, such as collision detection between robots and humans within varying postures encountered during the human–robot intersection. The experimental results demonstrate that RobotSDF can achieve high morphology accuracy up to the millimeter level and exhibit remarkable collision detection efficiency at the centisecond level even for obstacles in complex shapes, such as the human body.

RobotSDF is well-suited for precision manipulation tasks performed in confined environments. As a practical application, it can serve as a collision detector. Furthermore, its SDF prediction ability regarding obstacles can be integrated with the artificial potential field method in order to guide the robot to avoid directions that reduce the distance to obstacles, enabling real-time dynamic path planning. Since the precise locations of query points are difficult to obtain in an industrial environment, an image of the working environment can be involved with RobotSDF in future research.

## Figures and Tables

**Table 1:** Accuracy evaluation for each JointSDF.

Name	JointSDF1	JointSDF2	JointSDF3	JointSDF4	JointSDF5	JointSDF6
RMSE (std) (mm)	8.11 (7.58)	5.13 (3.17)	6.53 (4.04)	5.66 (4.05)	6.31 (4.30)	6.52 (4.57)

**Table 2:** Average RMSE comparison between the proposed RobotSDF and other systems.

	Robot Type	RMSE (mm)	Std (mm)
RobotSDF (Ours)	Elfin E03 with Gripper	6.38	4.62
Neural-JSDF [22]	Franka Emika Panda	10.40	7.80
CDF [44]	Franka Emika Panda	18.90	8.00

**Table 3:** Collision detection results for 20 experimental scenarios.

	**S1**	**S2**	**S3**	**S4**	**S5**	**S6**	**S7**	**S8**	**S9**	**S10**
SAT	Y	N	Y	N	N	Y	N	N	N	Y
GJK	Y	N	N	N	N	N	N	N	N	N
RobotSDF	N	N	N	N	N	N	N	N	N	N
GT	N	N	N	N	N	N	N	N	N	N
	**S11**	**S12**	**S13**	**S14**	**S15**	**S16**	**S17**	**S18**	**S19**	**S20**
SAT	Y	Y	Y	Y	Y	Y	Y	Y	Y	Y
GJK	Y	Y	Y	Y	Y	Y	Y	Y	Y	Y
RobotSDF	Y	Y	Y	Y	Y	Y	Y	Y	Y	Y
GT	Y	Y	Y	Y	Y	Y	Y	Y	Y	Y

## Data Availability

All data used for the experiments are publicly available at: https://github.com/YoungRainy/RobotSDF/tree/main (accessed on 10 July 2024).
